# Creatine Supplementation in Crohn’s Disease: Possible Therapeutic Trends in Symptoms and Muscle Tissue Integrity

**DOI:** 10.3390/muscles5040071

**Published:** 2026-10-04

**Authors:** Konstantinos Papadimitriou, Richard B. Kreider, Georgia-Eirini Deligiannidou, Constantinos Giaginis, Sousana K. Papadopoulou

**Affiliations:** 1 Department of Nutritional Sciences and Dietetics, School of Health Sciences, International Hellenic University, 57001 Thessaloniki, Greece; deligiannidoueirini@yahoo.gr (G.-E.D.); souzpapa@gmail.com (S.K.P.); 2 Exercise & Sport Nutrition Lab, Human Clinical Research Facility, Department of Health & Kinesiology, Texas A&M University, College Station, TX 77843, USA; rbkreider@tamu.edu; 3 Laboratory of Hygiene and Environmental Protection, School of Medicine, Democritus University of Thrace, Dragana, 68100 Alexandroupolis, Greece; 4 Department of Food Science and Nutrition, School of Environment, University of Aegean, 81100 Myrina, Greece; cgiaginis@aegean.gr

**Keywords:** inflammatory bowel disease, NHANES, clinical studies, foods

## Abstract

Background/Objectives: Creatine (Cr) supplementation may serve as a nutritional intervention, enhancing gut metabolism and preserving lean body mass, specifically fat-free mass, thereby improving functional capacity and quality of life in individuals with Crohn’s disease (CD). This paper aims to explore the potential mechanisms through which Cr supplementation may influence CD patients and review the current literature on this topic. Results: Studies were analyzed to assess the relationship between Cr supplementation and CD. Preliminary evidence suggests potential therapeutic benefits, although data remain limited. Discussion: Cr supplementation warrants further investigation through clinical trials and cohort studies. Emerging data from both in vitro and in vivo models indicate creatine monohydrate (CrM) may constitute a biologically reasonable supportive strategy, requiring formal evaluation in clinical trials in patients with CD. Conclusions: As a foundational step, further analysis of National Health and Nutrition Examination Survey (NHANES) data to document Cr-rich food sources and daily intake patterns is recommended before initiating clinical trials in Crohn’s disease cohorts.

## 1. Introduction

Creatine (Cr) is synthesized endogenously from the amino acids arginine, glycine, and methionine, primarily in the liver, kidneys, and pancreas [1]. The synthesis pathway involves two primary enzymes: glycine amidinotransferase and guanidinoacetate N-methyltransferase [2]. However, Cr delivered as a supplement, primarily as creatine monohydrate (CrM) (95% of clinical trials), is the most widely studied and commonly approved form of Cr for use in dietary supplements worldwide, owing to its well-established efficacy, safety, and bioavailability compared with other forms of Cr [3]. It has a pivotal role in energy homeostasis, particularly during high-intensity activities and metabolically stressful conditions (e.g., mitochondrial dysfunction, ischemia, etc.) [2]. The chemical structure of Cr comprises a methylguanidine acetic acid moiety, enabling it to readily accept a phosphate group. Energy is liberated from the degradation of the high-energy phosphate bond in phosphocreatine (CP), which is a naturally occurring Cr phosphorylated molecule. Through the creatine kinase (CK) reaction, CP-mediated phosphotransfer rapidly converts ADP back to ATP, thereby limiting transient increases in ADP near sites of ATP utilization. When CP availability decreases, the capacity of CK to buffer ATP and maintain ADP homeostasis is reduced, resulting in greater fluctuations in cellular ADP concentrations [4]. Practically, inside the muscles, this sustains muscle power and delays fatigue, especially in anaerobic alactic efforts (≈12 s) [5]. Moreover, it has been proven that this contributes to the treatment of many diseases (liver, kidney, cardiovascular, etc.) [6,7,8].

Creatine monohydrate (CrM) supplements have been extensive in athletes’ samples, depicting their beneficial role in performance, enhancing muscular strength, power [9], and high-intensity exercise capacity by facilitating ATP regeneration [10]. Specifically, its effect is particularly pronounced in activities involving short bursts of maximal effort, such as weightlifting, sprinting, and jumping and, in some cases, endurance (>~3 min in duration) [11]. Prolonged Cr ingestion (0.3 g/kg/day for 5–7 days and 0.03 to 0.15 g/kg/day thereafter) [12,13] induces an increase in fat-free mass [14], stimulating protein synthesis and potentially leading to increased muscle mass and strength gains over time [15]. Consequently, there is an increasing number of studies that utilize CrM supplements in the general population, from infants to the elderly, seeking to augment their Cr stores and, by extension, lean body mass. Of course, its beneficial role extends to factors such as injury and neurocognitive disorder prevention, neuroprotection (concussion, traumatic brain injury (TBI), spinal cord injuries), and improvements in cognitive function, pointing to Cr’s necessity throughout the lifespan [13]. Most studies show Cr is helpful for neuromuscular diseases affecting muscle, but not if neural damage has occurred [16]. For this reason, many authors are searching for Cr’s beneficial influence on pathological conditions [16] or, on rare occasions, the side effects [17,18].

Cr has been shown to play a crucial role in preserving intestinal mucosal integrity and cellular energy during stress conditions [19]. In rodent models, Cr treatment maintained barrier integrity, reduced oxidative injury, and preserved electrophysiological properties compared to the control group [20]. Genetic studies have revealed that mutations in the Glycine Amidinotransferase (GATM) gene, which is involved in Cr biosynthesis, lead to increased susceptibility to colitis, while Cr supplementation reverses the damage [21]. Cr also supports epithelial cell metabolism and tight junction integrity through its Cr transporter, which was found to be reduced in patients with inflammatory bowel disease (IBD) [19]. Overall, Cr’s ability to regulate energy balance and barrier function underscores its therapeutic potential in both organ preservation and intestinal diseases, and by extension, probably in pathological conditions such as Crohn’s disease (CD).

In general, IBD encompasses a complex and severe group of gastrointestinal disorders characterized by chronic or relapsing inflammation, which significantly affects patients’ overall health, quality of life, and healthcare costs [22]. The two principal forms of IBD are CD and Ulcerative Colitis (UC), which are distinguished by their distinct anatomical involvement and histopathological patterns [23]. Specifically, CD can affect any part of the gastrointestinal tract, from the mouth to the colon, causing mucosal inflammation and ulceration. During periods of disease exacerbation, persistent and dysregulated immune activation may impair normal tissue repair and mucosal healing, thereby contributing to the maintenance and progression of intestinal lesions [24].

Although the precise etiology of CD remains uncertain, characterizing it as an autoimmune disease, it is widely recognized as a multifactorial condition involving genetic susceptibility, dysbiosis of the gut microbiota, and various environmental influences [25]. Accumulating evidence suggests that dietary patterns, particularly those associated with a Western diet, can adversely affect the gut microbiome, immune responses, and mucosal integrity, thereby contributing to the pathogenesis of IBD [26,27]. In the context of nutritional status, patients with CD are particularly vulnerable to malnutrition due to factors such as inadequate dietary intake, nutrient malabsorption, increased gastrointestinal losses, and medication-related side effects [28,29]. The degree of malnutrition in CD patients varies, depending on the extent and severity of intestinal involvement and other contributory factors [30].

Consequently, malnutrition may lead to reductions in muscle mass and function. This raises the question of whether Cr supplementation could serve as a nutritional intervention aimed at enhancing gut metabolism, maintaining lean body mass specifically through the preservation or augmentation of fat-free mass, thereby improving both the functional capacity and quality of life in individuals with CD. The present narrative review seeks to explore the potential mechanisms through which creatine supplementation may benefit CD patients’ symptoms and muscle tissue and to review the current body of studies on this topic.

## 2. Narrative Review Construction

The present narrative review was structured according to established principles for the development of narrative reviews, with particular attention paid to the organization of the manuscript, the relevance of the selected literature, and integration of evidence from clinical, preclinical, and mechanistic studies. Given the limited number of clinical studies directly investigating Cr supplementation in patients with CD, a broad narrative approach was adopted to integrate evidence concerning Cr metabolism, intestinal barrier function, inflammation, skeletal muscle metabolism, and CD. The studies were searched from January of 2000 until March of 2026.

### Literature Search and Study Selection

A comprehensive literature search was conducted using the PubMed, Scopus, and Google Scholar electronic databases. The search focused primarily on studies examining the relationship between Cr metabolism or supplementation and CD. The search strategy used combinations of terms including “creatine” OR “creatine supplementation” OR “creatine monohydrate” OR “phosphocreatine” AND “Crohn’s disease” OR “inflammatory bowel disease” OR “intestinal inflammation” OR “intestinal barrier” OR “intestinal epithelial cells”. Additional searches combined creatine-related terms with “creatine transporter” OR “creatine kinase” OR “mucosal integrity” OR “gut metabolism”. To examine the potential role of creatine in preserving skeletal muscle in CD, supplementary searches included combinations of “Crohn’s disease” OR “inflammatory bowel disease” with “sarcopenia”, “muscle mass”, “muscle wasting”, “muscle protein synthesis”, “mTOR”, “IGF-1”, and “gut–muscle axis”.

Considering the scarcity of clinical trials specifically examining Cr supplementation in CD, no restriction to randomized controlled trials was applied. Eligible evidence therefore included clinical studies, observational studies, case reports, narrative and systematic reviews, as well as relevant in vitro and in vivo experimental studies. Preclinical studies were considered particularly relevant when they provided mechanistic information concerning Cr metabolism, intestinal epithelial energy homeostasis, creatine transporter activity, mucosal integrity, or inflammatory responses.

Studies were considered for inclusion when they contributed directly to at least one of the major objectives of the review: (i) describing mechanisms contributing to skeletal muscle degradation in CD; (ii) explaining the anabolic and bioenergetic effects of Cr on skeletal muscle; (iii) examining existing evidence concerning Cr supplementation in CD; (iv) investigating the role of the phosphocreatine–creatine system, creatine kinase, CrT1/SLC6A8, and HIF signaling in intestinal barrier function; or (v) identifying potential directions for future clinical investigation of Cr supplementation in CD. Articles that were unrelated to these objectives or did not provide relevant mechanistic, clinical, or nutritional information were excluded.

## 3. The Mechanisms of Muscle Degradation in Crohn’s Disease

According to Nardome et al.’s [31] review, muscle mass degradation in CD results from the interaction of chronic intestinal inflammation, gut dysbiosis, malnutrition, and reduced physical activity through the proposed gut–muscle axis [32,33]. Persistent intestinal inflammation increases circulating levels of pro-inflammatory cytokines, particularly tumor necrosis factor-alpha (TNF-α) and interleukin-6 (IL-6), which activate inflammatory and catabolic signaling pathways, including nuclear factor kappa B (NF-κB), Janus kinase/signal transducer and activator of transcription (JAK/STAT), and mitogen-activated protein kinase (MAPK) pathways [34]. Chronic inflammatory signaling may also impair the anabolic phosphoinositide 3-kinase/protein kinase B/mammalian target of the rapamycin (PI3K/Akt/mTOR) pathway, thereby reducing muscle protein synthesis. Collectively, these alterations may enhance proteolytic signaling, including the upregulation of the muscle-specific E3 ubiquitin ligases MuRF1 and Atrogin-1, ultimately contributing to accelerated skeletal muscle loss [35]. In parallel, inflammatory mediators and bacterial lipopolysaccharides (LPS) induce oxidative stress and mitochondrial dysfunction, further impairing ATP production and muscle metabolism [36].

Also, they are observed alterations in the gut microbiota that reduce beneficial short-chain fatty acid (SCFA)-producing bacteria, increasing intestinal permeability and systemic inflammation while weakening anabolic pathways involved in muscle maintenance [37,38]. At the same time, malabsorption, reduced dietary intake, increased energy expenditure, and deficiencies of protein, vitamin D, and other micronutrients promote muscle catabolism and anabolic resistance [39]. In experimental animal models, these mechanisms are supported, demonstrating reduced muscle mass, decreased Insulin-like Growth Factor 1 (IGF-1)/mTOR signaling, and increased expression of Muscle Ring Finger Protein 1 (MuRF1), Atrogin-1, and myostatin in response to intestinal inflammation [33]. Together, these interconnected processes drive progressive skeletal muscle degradation in patients with CD.

## 4. The Anabolic Mechanisms of Creatine Supplementation in Muscle Tissue

Cr is one of the most extensively investigated ergogenic aids and is widely recognized for its capacity to enhance skeletal muscle mass, strength, and exercise performance, particularly when combined with resistance training [40]. More than 90% of the body’s Cr stores are located in skeletal muscle, where Cr and phosphocreatine contribute to maintaining cellular energy homeostasis by facilitating rapid ATP regeneration during muscular contractions [41]. Beyond its well-established bioenergetic role, increasing evidence suggests that Cr supplementation exerts pleiotropic effects on skeletal muscle metabolism, contributing to muscle hypertrophy, improved recovery, and potential therapeutic benefits in clinical populations [42]. Despite the consistent demonstration of its anabolic effects, the precise molecular mechanisms through which Cr promotes skeletal muscle growth remain incompletely understood.

Several complementary mechanisms have been proposed to explain the hypertrophic effects of Cr supplementation. Early investigations suggested that the increase in intracellular Cr concentration induces osmotic cell swelling, creating a mechanical and metabolic stimulus that promotes anabolic signaling and protein synthesis [43,44]. More recent evidence indicates that Cr may directly activate key regulators of muscle protein synthesis, including the mTOR signaling pathway, thereby enhancing translational efficiency and promoting muscle accretion. Furthermore, Cr supplementation influences the expression of anabolic growth factors and myokines, including IGF-1 [45], while reducing the expression of myostatin, a potent negative regulator of muscle growth [46]. These molecular adaptations are accompanied by increased satellite cell proliferation, enhanced myogenic differentiation, and improved muscle regeneration, collectively contributing to greater skeletal muscle hypertrophy.

Skeletal muscle hypertrophy is ultimately determined by the balance between muscle protein synthesis and muscle protein breakdown, making interventions that favor a positive protein balance particularly relevant for athletes and clinical populations [47]. Current evidence indicates that Cr supplementation supports this anabolic environment through multiple interconnected mechanisms, including increased phosphocreatine availability, enhanced training capacity, activation of mTOR signaling, elevated intracellular hydration, stimulation of satellite cell activity, reduced exercise-induced muscle damage, and improved post-exercise recovery [48]. Rather than acting through a single pathway, Cr appears to exert synergistic effects on cellular bioenergetics, anabolic signaling, and myogenesis, thereby amplifying the adaptive response to resistance exercise. Although additional mechanistic research is warranted, the available literature strongly supports Cr supplementation as an effective nutritional strategy for promoting skeletal muscle growth and optimizing muscle protein metabolism. Furthermore, studies investigating the potential side effects of Cr have generally demonstrated a favorable safety profile. To date, no clinically relevant interactions with biologic therapies, corticosteroids, immunomodulators, mesalazine, antibiotics, or dietary interventions have been reported in studies examining the potential beneficial effects of Cr [49,50]. Nevertheless, caution is warranted, particularly in populations that have not been extensively studied and in cases involving high doses of Cr [51].

## 5. Creatine Supplementation and CD

Unique scientific data regarding Cr supplementation in individuals with CD have been reported in a case study involving a 33-year-old patient who received 1.034 g/day of creatine hydrochloride (CrHCl) [52]. However, Kreider, Jäger, and Purpura [2] concluded that CrHCl does not offer superior absorption or muscle Cr retention compared to CrM. Also, the manufacturers of CrHCl claimed that CrHCl is more bioavailable; however, this was without substantiated clinical evidence. Furthermore, the review highlighted that CrHCl does not outperform CrM in enhancing physical performance, hormonal responses, or body composition. As a result, the researchers classified CrHCl as having limited evidence supporting its efficacy compared to a placebo.

In the case study, the authors do not clearly describe how the patient decided to initiate supplementation, nor do they state whether a physician prescribed it [52]. Notably, Cr’s potential beneficial effects were observed only after four months, during which the patient discontinued Cr supplementation and began treatment with mesalamine (Salix Pharmaceuticals, Raleigh) at a dose of 1.5 g/day for the treatment of Crohn’s ileitis. During this time, the patient’s symptoms worsened, characterized by progressively more frequent and severe abdominal pain and extensive ulceration, ultimately leading the patient to resume Cr supplementation. Upon resumption, symptom remission was reported [52].

Nevertheless, even during the period before discontinuing Cr, the patient had a two-year history of hematochezia and anal pain thought to be secondary to non-healing anal fissures, as well as rectal bleeding, chronic cramping abdominal pain, and loose stools. Thus, two hypotheses can be proposed, considering the statements from the case study: (a) long-term supplementation with CrHCl contributed to the manifestation of the two-year history of symptoms, and (b) the patient’s adaptation to 1.034 g/day of CrHCl established a stable condition, suggesting that Cr supplementation contributed to symptom remission.

## 6. How Creatine Would Contribute to CD Patients’ Muscle Integrity and Symptom Remission?

Skeletal muscle loss represents an important extraintestinal consequence of CD, arising from the complex interaction of chronic intestinal inflammation, gut dysbiosis, malnutrition, and reduced physical activity through the proposed gut–muscle axis [31,32]. These interconnected disturbances may impair skeletal muscle energy metabolism, promote systemic inflammation, disrupt anabolic signaling, and shift muscle protein turnover toward a catabolic state [37,38]. In this context, Cr supplementation may represent a biologically plausible nutritional strategy, given its established role in maintaining cellular energy homeostasis through rapid ATP regeneration [40,41] and its potential influence on mechanisms involved in muscle protein synthesis, hypertrophy, and resynthesis [42,43,45,46,47,48]. Therefore, considering both the mechanism’s underlying CD-associated symptoms and the anabolic and bioenergetic actions of Cr provides a rationale for exploring whether Cr supplementation could contribute to the preservation of skeletal muscle mass and function in patients with CD.

Studies on CD and macro- and micronutrient consumption have indicated that imbalances in food intake may be positively correlated with the manifestation of symptoms [53]. This suggestion was first introduced in a case report involving a 22-year-old elite male endurance athlete, who, despite the absence of a family history of CD, unlike the 33-year-old patient, was noted to have unbalanced dietary habits, characterized by a high-fat diet and a lack of micronutrients [54].

In the current case, it is plausible that excessive Cr supplementation (1.034 g/day) combined with an unknown dietary pattern created an imbalanced intestinal environment, leading to gut microbiota dysbiosis [55], degradation of the mucosal layer, and subsequent symptom manifestation [24]. On the other hand, two scientific reports have indicated that Cr may inhibit colon cancer growth [56,57], suggesting a potential role for Cr in modulating inflammatory processes associated with CD. Additionally, Li and Yang [58] revealed that Cr supplementation enhances antitumor T cell immunity in multiple preclinical mouse tumor models, improving antitumor efficacy.

Although there are contradictory reports, Gkikas et al. [55] propose a different explanation. Specifically, they support that dietary triggers act differently across populations, with the microbiome serving as a critical modifier of dietary influences on the development of IBD. This variability likely explains why the 33-year-old patient requested the resumption of Cr supplementation after four months. In other words, a systematic dietary imbalance (e.g., high intake of processed foods and red meat) can trigger gut microbiota dysbiosis and contribute to CD onset [53]. However, following this trigger phase, the organism may adapt to the altered environment, and efforts to return to a balanced diet might paradoxically exacerbate symptoms [55].

In CD, the muscle mass degradation has many theories and misconceptions, making it difficult for the authors to precisely depict its mechanisms. However, this multifactorial disease seems to be focused on the small intestine, observing the degradation of the mucosa’s layer structure. This degradation induces epithelial cell exposure to luminal bacteria [59]. The epithelial cells’ exposure causes injury in many parts of the intestine [60]. As a result, it is observed that intestinal dysregulation in food absorption causes insufficient energy intake [53,61] and consequently, macronutrient depletion in the blood circulation, inducing muscle atrophy [55]. However, how can Cr supplementation remiss or reverse this degradation mechanism?

Regarding Cr supplementation, CrM is widely considered the gold standard for its ergogenic benefits, with a strong safety profile [13]. Specifically, the CP shuttle system supports mucosal barrier function and wound healing by improving cellular energetics and stabilizing adherent junctions [62,63]. During active inflammation, such as that occurring in CD, reduced oxygen (O_2_) availability stabilizes hypoxia-inducible factors (HIFs), which promote the expression of CK isoenzymes and the Cr transporter 1 (CrT1), encoded by the X-linked solute carrier family 6-member 8 (SLC6A8) gene [62], in intestinal epithelial cells. CK localizes at epithelial junctions, where it interacts with the actin cytoskeleton and contributes to the maintenance of epithelial structural integrity. In the context of epithelial disruption, large amounts of ATP are required to support cytoskeletal reorganization, including acto-myosin ATPase activity at cellular junctions. Under these conditions, CK and CrT1 work coordinately to promote wound healing and barrier function by synthesizing ATP from PCr, thereby maintaining epithelial homeostasis [62,63,64].

In the case of the 33-year-old patient, CrHCL improved him clinically, and the prior extensive ulceration and inflammation in the terminal ileum appeared significantly improved, and only one small aphthous ulcer was seen. Observing the beneficial effects of Cr, the therapy was continued. However, what would happen in the case of the utilization of another more or equally effective Cr supplement, such as CrM, whose influence has been proven more or equally beneficial than other Cr types [1,64]?

A novel intraluminal preservation solution, tailored to address energy depletion and oxidative stress in stored intestines, was supplemented with Cr to improve mucosal integrity in rodent models. The intestinal tissue showed higher levels of Cr phosphate and ATP, preserved transepithelial resistance, and reduced permeability after 10 h of cold storage compared to untreated controls. Histological analysis revealed substantially less intestinal tissue damage in Cr-loaded samples, suggesting that enhancing cellular energy reserves may be a viable strategy for maintaining intestinal viability during transplantation or storage [20].

Additionally, complementary studies on the CrT/SLC6A8 in intestinal epithelial cells have shown its crucial role in maintaining energy homeostasis and barrier function. CrT, which localizes at tight junctions, was found to regulate intracellular Cr levels, wound healing, and epithelial integrity. Knockdown or deficiency of CrT led to impaired barrier formation and increased reliance on glycolysis, contributing to tight junction dysfunction and cellular stress. Moreover, mucosal biopsies from patients with IBD exhibited significantly reduced CrT expression, potentially linking Cr metabolism deficiencies to compromised barrier integrity in IBD [19]. These findings align with broader insights into dietary creatine’s role in human physiology, highlighting its importance for energy metabolism and gastrointestinal health [65].

**Figure 1:**
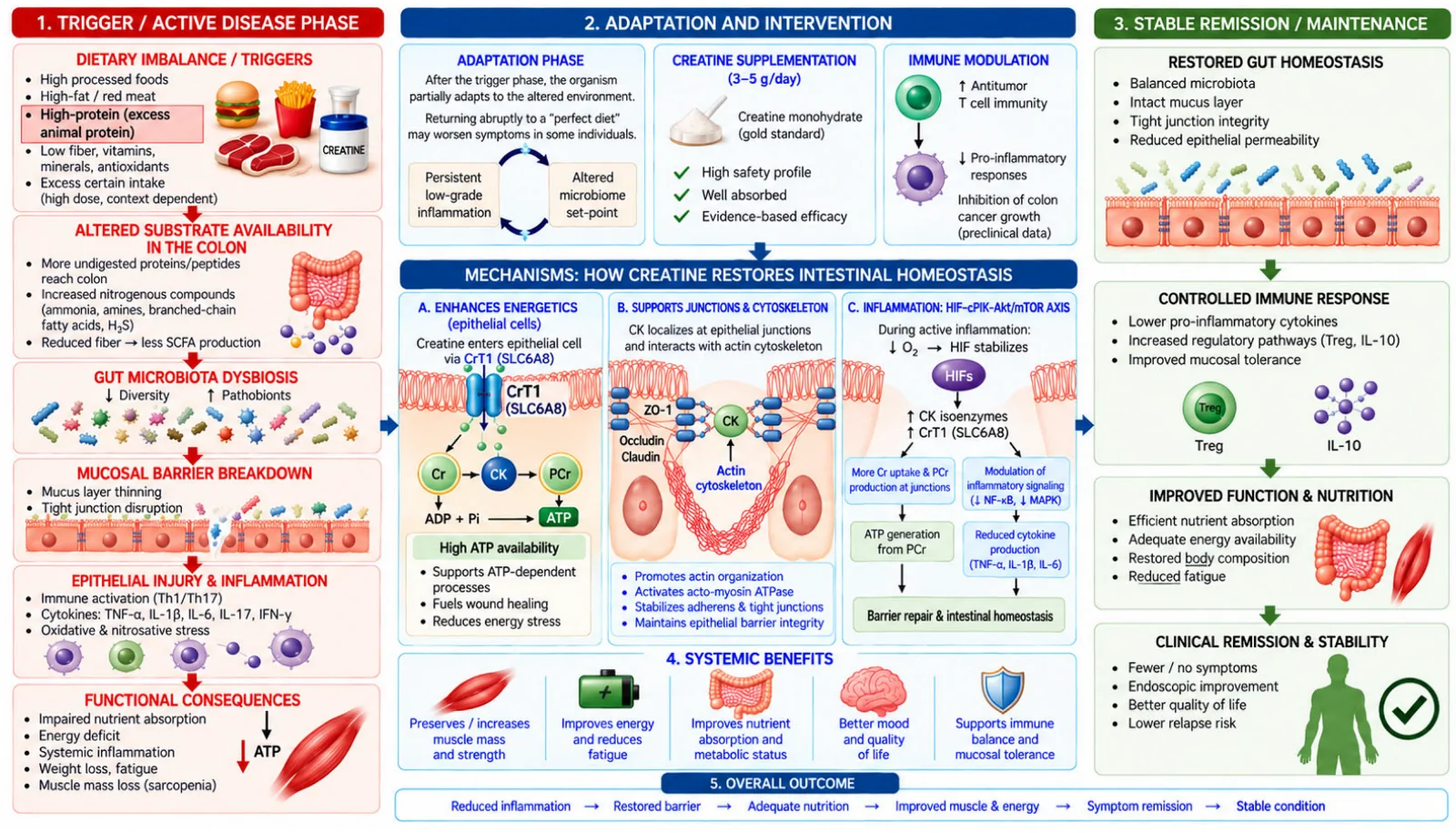
Schematic representation of the possible influence of Cr on CD symptoms. Th1: T helper 1 cell; Th17: T helper 17 cell; TNF-α: tumor necrosis factor alpha; IL-1β: interleukin-1 beta; IL-6: interleukin-6; IL-17: interleukin-17; IFN-γ: interferon gamma; ATP: adenosine triphosphate; CrT1: creatine transporter 1; SLC6A8: solute carrier family 6 member 8; Cr: creatine; CK: creatine kinase; PCr: phosphocreatine; ADP: adenosine diphosphate; ZO-1: zonula occludens-1; HIF: hypoxia-inducible factor; PI3K: phosphoinositide 3-kinase; Akt: protein kinase B; mTOR: mechanistic target of rapamycin; NF-κB: nuclear factor kappa B; MAPK: mitogen-activated protein kinase; Treg: regulatory T cell; IL-10: interleukin-10.

Roy & Lee [52], in their case study, noted that in future studies, using 3–5 g of chemically pure CrM per day, given for 3–6 months either as a monotherapy or in combination with low dose metformin (0.5–1.0 g per day), would potentially be able to improve several quality-of-life parameters of patients with IBD. Considering the wide spectrum of beneficial effects of Cr, with improvement in muscle mass and strength, mobility, and vital functions, and less fatigue and depression [66], its utilization seems important. However, since 1932, when Burrill B. Crohn [66] first published his report on CD (Crohn, Ginzburg, Oppenheimer, 1932) [67], there have not been well-studied protocols. It is possible that, because the last decay has been answered more scientifically, we can avoid any severe side effects after Cr supplementation [13]. In Figure 1, we represent, considering our findings, how Cr probably influences CD symptoms.

## 7. How Can We Begin the Research Journey of Creatine Contribution?

The present topic highlights the limited available evidence and the lack of clinical and cohort studies that could contribute to determining the potential effects of Cr on CD symptoms. However, it is well established that certain foods are notable for their Cr content (g/kg), including herring (6.5–10), beef (4.5), salmon (4.5), pork (5), cod (3), tuna (4), milk (0.1), cranberries (0.02), and shrimp (0.7) [53]. The cooking method and temperature should also be considered, as they may influence the Cr content of foods [68]. Available evidence indicates that baking results in the highest Cr retention, followed by frying and boiling, although the differences among cooking methods were not statistically significant. In addition, brining does not appear to significantly affect the retention of these components after cooking [69].

According to the study by Papadimitriou et al. [53], foods most commonly associated with symptom relief and preferred by CD patients include yogurt, bananas, dairy products, rice, kefir, olive oil, potatoes, fish, chicken, vegetables, fruits, and meat (primarily pork). Notably, many of these items contain moderate to high levels of Cr. Therefore, the response to Cr may depend on habitual food intake; hence, there is an open window for searching strategies and the influence of Cr-rich foods on CD symptoms (Table 1).

Also, it is essential to recognize that patients with CD may benefit from consuming foods that are naturally high in Cr, focusing on plant-based diets that have many benefits, especially for those with high blood pressure, diabetes, cardiovascular disease, or obesity [70]. However, studies in CD need further investigations about the remission of disease symptoms [71]. A National Health and Nutrition Examination Survey (NHANES) would be a significant step for the recognition of CD patients’ daily Cr intake through their food consumption [67]. However, these suggestions must be considered alongside receiving personalized care from a dietitian experienced in IBD, especially in the cases of restrictive diets and interventions. Based on the cited studies, CD patients already prefer and tolerate many Cr-rich foods in their daily diet. Furthermore, considering Cr supplementation has been studied for its therapeutic potential in conditions such as sarcopenia [72], aging [73], cardiovascular disease, diabetes, neurodegenerative disorders, muscular dystrophy, and even in oncology [16], it is timely to explore its role in CD management.

Consistent with Roy & Lee’s [52] proposal, we also recommend assessing the expression of the CrT1 in intestinal epithelial cells via biopsy before prescribing Cr supplementation. This biomarker would serve as a critical parameter for determining the likelihood of treatment efficacy, as patients with active disease and significant ulceration may have impaired creatine absorption. Conversely, given Cr’s potential to accelerate tissue regeneration, it may offer a novel therapeutic avenue for inducing remission in CD or any other phenotype, such as patients with a mild form of ileal inflammation, small bowel resection, or active perianal disease, whose prevalence has increased markedly in recent decades.

## 8. Conclusions

Evidence from experimental colitis and intestinal epithelial models suggests that Cr-related pathways may contribute to the maintenance of intestinal barrier and muscle tissue integrity. However, whether these mechanisms translate into clinically meaningful benefits in patients with CD remains unknown and requires investigation in appropriately designed clinical studies. Despite the current lack of CD-specific evidence, recent findings from both in vitro and in vivo models provide a biological rationale for investigating whether Cr may contribute to the management of CD-related symptoms. Furthermore, dietary studies, including analyses based on NHANES data, could provide valuable information regarding the consumption of creatine-rich foods and patterns of daily Cr intake. Similar approaches should also be applied specifically to CD cohorts to investigate the potential association between dietary Cr intake and CD symptoms.

## Figures and Tables

**Table 1:** Foods that CD patients consume and their creatine content.

Foods	Creatine Content (g· kg^−1^ Mass)
Rice	≈0.32
Potatoes	≈0.18
Salmon	≈4.5
Chicken	≈0.8
Meat (beef, pork)	≈4.5–5

## Data Availability

No new data were created or analyzed in this study. Data sharing is not applicable to this article.

## Abbreviations

The following abbreviations are used in this manuscript:

Cr	Creatine
CD	Crohn’s disease
NHANES	National Health and Nutrition Examination Survey
CP	Phosphocreatine
CK	Creatine kinase
ATP	Triphosphoric adenosine
ADP	Diphosphoric adenosine
CrM	Creatine monohydrate
TBI	Traumatic brain injury
StO_2_	Oxygen saturation
Gatm	Glycine aminotransferase
IBD	Inflammatory bowel disease
UC	Ulcerative colitis
TNF-α	Tumor Necrosis Factor-alpha
IL-6	Interleukin-6
NF-κB	Nuclear Factor kappa-light-chain-enhancer of activated B cells
JAK/STAT	Janus Kinase/Signal Transducer and Activator of Transcription
MAPK	Mitogen-Activated Protein Kinase
PI3K	Phosphoinositide 3-Kinase
mTOR	Mechanistic Target of Rapamycin
MuRF1	Muscle RING Finger Protein 1
LPS	Lipopolysaccharide
SCFA	Short-Chain Fatty Acid
IGF-1	Insulin-like Growth Factor 1
CrHCL	Creatine hydrochloride
O_2_	Oxygen
HIF	Hypoxia-inducible transcription factors
CrT1	Creatine transporter
SLC6A8	Solute carrier family 6 member 8
